# Symptoms from Morphine

**Published:** 1895-12

**Authors:** Rufus L. Thurston

**Affiliations:** Boston, Mass.


### SYMPTOMS FROM MORPHINE.

RUFUS L. THURSTON, M. D., BOSTON, MASS.

The following symptoms were developed in a woman who took one grain of Morphine for sleeplessness :

Widow, aet. twenty-six years, red hair, spare, a tall, nervous temperament. Slept soundly all night after taking the drug, but the symptoms began the next morning on waking and continued for about four days.

*Mind.*—Irritable ; wants to be alone.

*Head.*—Dull pain ; brain feels tense, as though wound up tightly ; vertigo, *from least motion of head* (two days).

*Eyes.*—Sight dim, " blurred."

*Stomach.*—Vomiting of bitter, greenish water.

*Abdomen.*—Distended.

*Extremities.*—Wandering sharp pains in legs and feet ; numbness of legs and feet, with falling on attempting to stand.

*Generalities.*—Purple spots on body ; chills with rigors ; *sudden* attacks of fainting (several times daily for four days).

